# Plasma ctDNA kinetics as a predictor of systemic therapy response for advanced non-small cell lung cancer: a systematic review and meta-analysis

**DOI:** 10.1093/oncolo/oyae344

**Published:** 2025-02-25

**Authors:** Luís F Leite da Silva, Erick F Saldanha, Júnior Samuel Alonso de Menezes, Leonardo Halamy Pereira, João Alexandre R de Bragança dos Santos, Isabella Romagnoli Buonopane, Erito M de Souza, Caio Ulysses Galvani de Menezes, Gilberto Lopes

**Affiliations:** Departmento de Ciências Médicas, Universidade Federal Fluminense, Niterói, RJ 24033-900, Brazil; Department of Medical Oncology and Hematology, Princess Margaret Cancer Centre, University Health Network, University of Toronto, ON M5G 2M9, Canada; Departamento de Ciências da Saúde, Universidade Federal da Bahia, Salvador, BA 21941-590, Brazil; Departmento de Ciências Médicas, Universidade Federal Fluminense, Niterói, RJ 24033-900, Brazil; Departmento de Ciências Médicas, Universidade Federal Fluminense, Niterói, RJ 24033-900, Brazil; Departamento de Ciências da Saúde, Universidade Federal da Bahia, Salvador, BA 21941-590, Brazil; Departmento de Ciências Médicas, Universidade Federal Fluminense, Niterói, RJ 24033-900, Brazil; Departamento de Oncologia Clínica, Universidade Federal de São Paulo, SP 04023-062, Brazil; Sylvester Comprehensive Cancer Center, University of Miami, Miami, FL 33136, United States

**Keywords:** NSCLC, ctDNA, precision oncology, molecular response

## Abstract

**Background:**

Predicting early treatment response in advanced non-small cell lung cancer (NSCLC) is challenging. Longitudinal monitoring of circulating tumor DNA (ctDNA) can track tumor response to treatments like immune checkpoint blockade (ICB) and correlate with outcomes. This meta-analysis evaluated whether ctDNA clearance or decrease is associated with improved survival across various settings in NSCLC.

**Methods:**

A systematic review of MEDLINE, EMBASE, and Cochrane databases (up to April 2024) identified studies evaluating the impact of ctDNA kinetics on survival outcomes in non-curative NSCLC settings. Pooled hazard ratios (HR) for progression-free survival (PFS) and overall survival (OS) were calculated using a random effects model.

**Results:**

We included 32 studies with 3047 NSCLC patients receiving systemic therapies such as targeted therapy (TT), ICB, and chemotherapy. Meta-analysis of 31 studies showed that ctDNA decrease/clearance was linked to improved PFS (HR: 0.32 [0.26, 0.40], *I*² = 63%, *P* < .01). Subgroup analysis indicated strong PFS benefits from ctDNA clearance (HR: 0.27 [0.20, 0.36]). Similar improvements were seen across patients undergoing targeted therapy (HR: 0.34) and ICB (HR: 0.33). Analysis of 25 studies revealed a significant association between ctDNA reduction and better OS (HR: 0.31 [0.23, 0.42], *I*² = 47%, *P* < .01). Subgroup findings were consistent for both TT (HR: 0.41) and ICB (HR: 0.32). Sensitivity analysis demonstrated that ctDNA clearance/decrease was consistently associated with improved PFS across study designs and ctDNA analysis methods. There was no significant variation in hazard ratios for PFS based on NSCLC subtypes, smoking status, or sex.

**Conclusion:**

Plasma ctDNA kinetics was associated with improved survival outcomes in patients diagnosed with advanced NSCLC undergoing treatment with TT and ICB.

**Implications for clinical practice**

The results of this meta-analysis underscore the clinical relevance of longitudinal plasma ctDNA assessment in predicting survival for patients with advanced NSCLC. Integrating ctDNA kinetics analysis into routine clinical practice is a promising non-invasive biomarker that can aid clinicians in timely therapeutic decisions for patients with advanced NSCLC.

## Introduction

Non-small cell lung cancer (NSCLC) is the leading cause of cancer-related morbidity and mortality worldwide.^1,2^ Over the past decade, the treatment landscape of NSCLC has markedly changed due to a better understanding of the molecular features and the incorporation of targeted therapies (TT), immune checkpoint blockade (ICB), and novel therapeutics such as newer generations of tyrosine kinase inhibitors (TKIs) and antibody-drug conjugates (ADC), leading to higher survival rates and a shift toward personalization of treatment.^3,4^ Hence, a fast-evolving treatment armamentarium is available for patients with NSCLC in the earlier and advanced setting.^5,6^

Significant strides achieved in the personalized management of advanced NSCLC patients underscore the need for novel biomarkers to be developed and implemented for this patient population.^7^ Nonetheless, despite some patients presenting durable responses to therapy, most patients with metastatic NSCLC will not respond or experience short-lived responses due to treatment resistance.^8^ Hence, identifying patients who would benefit from treatment is critical. To this end, recent evidence supports liquid biopsy evaluation of circulating cell-free tumor DNA (ctDNA) as a promising biomarker that can track early tumor response to treatment.^9,10^

Plasma detection of ctDNA enables a less invasive blood-based approach, allowing for the detection of somatic alterations and a feasible method for serial measurements that can capture tumor evolution and complement tissue sampling.^11-15^ In NSCLC, highly sensitive ctDNA assays have been widely adopted in the routine clinical setting for cancer genotyping and identifying treatment resistance to targeted agents.^16-18^ Likewise, in the metastatic setting, evidence has shown that longitudinal ctDNA evaluation can track tumor burden dynamics and capture early-on-therapy responses.^19^ For instance, metastatic NSCLC patients treated with ICB presenting a reduction in the ctDNA levels demonstrated significantly longer survival outcomes compared to patients with no evidence of molecular response.^20^ A similar association was shown for patients treated with other therapeutic agents.^21-23^ Collectively, recent data suggests that ctDNA may be leveraged as a biomarker to predict therapeutic response and inform treatment decisions.^24^

We conducted a systematic review and meta-analysis to evaluate the role of plasma ctDNA kinetics in therapeutic response prediction for patients with advanced NSCLC undergoing systemic treatment.

## Material and methods

This systematic review was conducted following PRISMA guidelines^25^ and has been registered in PROSPERO (International Prospective Register of Systematic Reviews, CRD42024534489). Studies were analyzed to assess the impact of plasma ctDNA kinetics, including ctDNA clearance, decrease, or molecular response on survival endpoints.

### Search strategy and selection criteria

A comprehensive digital search was conducted from database inception to March 2024 across Embase, MEDLINE with the PubMed interface, and the Cochrane Central Register. The search strategy included the following terms: “Non-Small Cell Lung Cancer,” “NSCLC,” “Circulating Tumor DNA,” “ctDNA,” “liquid biopsy,” “ctDNA clearance,” “longitudinal ctDNA,” “Survival,” “overall survival,” “OS,” “progression-free survival,” “PFS,” and “progression-free survival.” We also screened previous meta-analyses on the topic to identify additional studies, and the references of eligible studies were evaluated during the screening process. Two investigators (L.F., J.M.) independently screened titles, abstracts, full texts, Supplementary Materials, online appendices, and reference lists for eligibility.

Inclusion criteria were restricted to studies involving: (1) patients with a confirmed diagnosis of NSCLC; (2) undergoing systemic therapy; (3) with longitudinal assessment of plasma ctDNA; (4) observational studies and clinical trials. Exclusion criteria excluded: (1) NSCLC patients in a curative treatment setting; (2) studies that did not correlate ctDNA changes with survival outcomes; (3) conference abstracts, case reports, letters, and reviews. There were no language restrictions for publication.

### Endpoints and subanalyses

The endpoints of this meta-analysis were overall survival (OS), defined as the duration from treatment initiation or diagnosis to death from any cause, and progression-free survival (PFS), defined as the duration from treatment initiation until disease progression or death from any cause. Prespecified subanalyses were conducted to investigate specific factors impacting overall estimates, including the type of therapy administered (chemotherapy, immunotherapy, targeted therapy, or combinations), timing of ctDNA evaluation relative to treatment initiation, and the method of liquid biopsy evaluation (tumor informed or tumor agnostic assays).

### Data extraction and quality assessment

Two authors performed data extraction using a predefined spreadsheet encompassing information regarding study design, patient characteristics, publication year, ctDNA analysis method (next-generation sequencing or polymerase chain reaction), clearance definition, ctDNA extraction timepoints, survival outcome definitions, and other characteristics. Tumor-informed assays, which are tailored to a patient’s specific tumor mutations and require available tissue samples, generally offer higher sensitivity compared to tumor-agnostic assays, which use broad mutation panels and are a “plasma-only” method, as a tissue sample is not necessary. NGS-based methods allow for broader mutation detection with higher sensitivity, while PCR-based methods provide higher specificity but are limited to detecting predefined mutations. These variations in detection methods and assay types could influence the sensitivity and outcomes reported in the studies. Subgroup analyses were performed to assess the impact of these detection methods on survival estimates.

Disagreements were resolved through consensus and, if unresolved, discussed with the senior author. The Quality in Prognostic Studies (QUIPS) tool^26^ recommended by Cochrane was implemented to evaluate the risk of bias. Using this tool, 2 investigators (J.S., L.H.) examined 5 different domains: (i) study participation, (ii) attrition, (iii) prognostic factor measurement, (iv) confounding factors, and (v) statistical analysis.

### Statistical analysis

The hazard ratio (HR) for OS and PFS, accompanied by 95% CI, was pooled using inverse-variance methods. To account for heterogeneity among studies, we also applied the DerSimonian and Laird random-effects model to estimate.^27^ Statistical heterogeneity was assessed using the Cochran *Q* test, quantified by the *I*^2^ statistic and Tau-square using the restricted maximum-likelihood estimator. Inter-study variability was assessed through the *I*^2^ metric and Cochran’s *Q* test, classifying values below 25% as low, between 25% and 50% as moderate, and above 50% as high heterogeneity.^28^

Sensitivity analyses were conducted to scrutinize potential sources of heterogeneity and ascertain the robustness of the primary findings. A separate pooling of clinical trials and observational studies was performed to investigate the potential impact of study design on overall hazard ratios. Different ctDNA assessment timepoints and assays were analyzed to further explore the heterogeneity of studies. Meta-regressions were employed to explore the impact of patient baseline characteristics on pooled estimates. Publication bias was evaluated through a contour-enhanced funnel plot^29^ to assess similar distributions of studies with similar weights and quantified with the Egger test.^30^

## Results

### Study selection and characteristics

**Figure 1.**
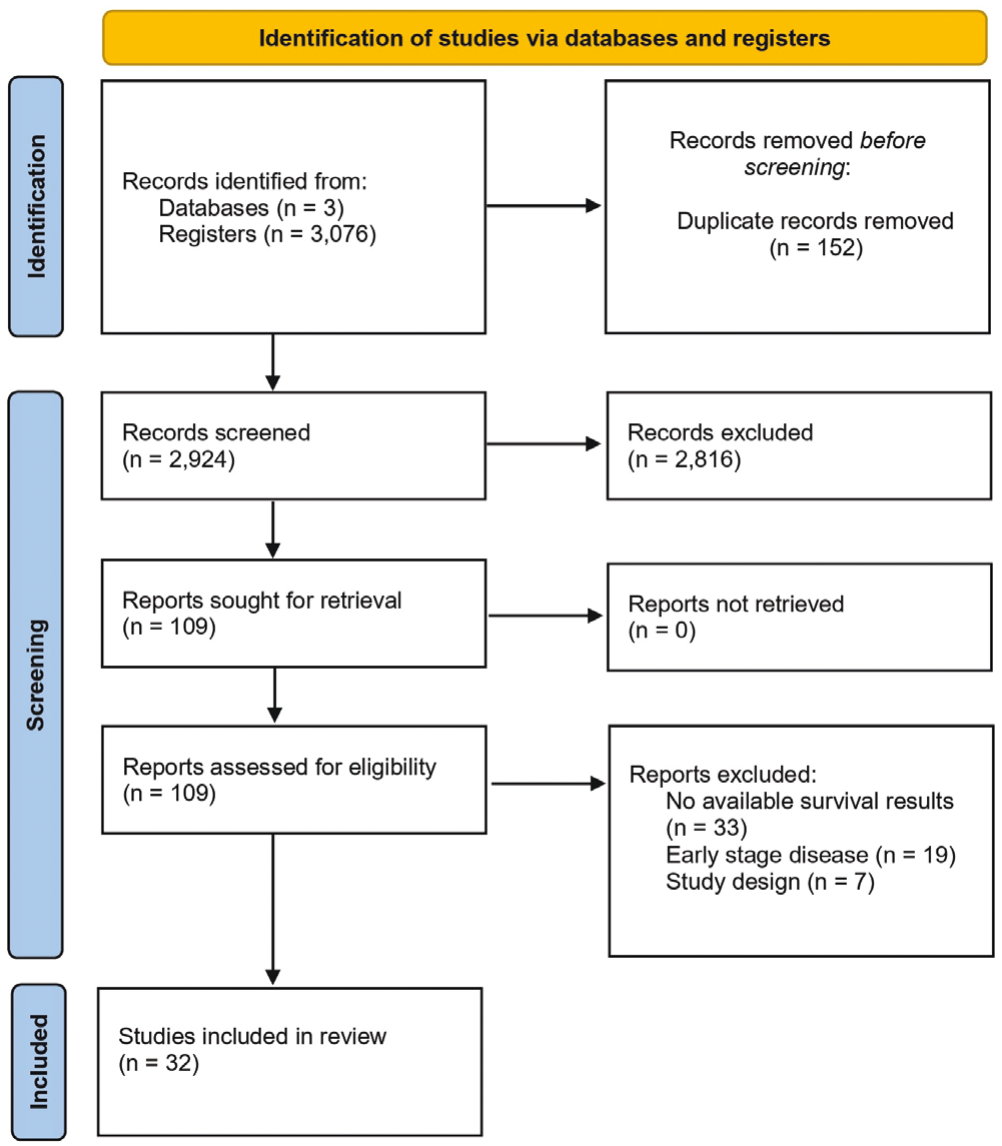
Prisma flowchart. Preferred reporting Items for systematic reviews and meta-analyses (PRISMA) flow diagram for literature search and selection.

As detailed in Figure 1, the initial search yielded 3076 results. After the removal of duplicate records and ineligible studies, 110 remained and were fully reviewed based on inclusion criteria. Of these, a total of 32 studies were included, comprising 3047 patients with NSCLC with longitudinal ctDNA assessment from 14 clinical trials, 12 non-randomized prospective cohorts, and 6 retrospective studies (ie, the latter two categories consisted of observational data).^9,20,21,31-57^ A total of 1940 (64%) patients received targeted therapies, encompassing *EGFR*, *ALK,* and *MET* TKIs, while 826 (27%) were treated with ICB and 281 (10%) patients had undergone chemotherapy without involving targeted treatments or ICB (Table 1). In most studies, baseline ctDNA was assessed immediately prior to the initiation of treatment, and sequential liquid biopsies were performed in the following weeks to monitor ctDNA kinetics. Included studies reported ctDNA clearance as the presence of undetectable mutations in plasma, while ctDNA decrease definitions varied across 30% or 50% reduction of VAF (Supplementary Table S1).

**Table 1.** Characteristics of studies associating longitudinal ctDNA assessment with advanced NSCLC survival.

First Author/year	Study design	NSCLC Driver mutation	Number of patients	Treatment	ctDNA detection method
Anagnostou, 2019	Prospective cohort	n/a	24	ICB	NGS, tumor informed
Anagnostou, 2023	Clinical trial	n/a	50	ICB	NGS, tumor agnostic
Buder, 2020	Retrospective cohort	*EGFR*	141	*EGFR TKI*	PCR, tumor informed
Ding, 2019	Clinical trial	*EGFR*	28	*EGFR TKI*	PCR, tumor informed
Duan, 2020	Clinical trial	*EGFR*	180	*EGFR TKI*	NGS, tumor informed
Ebert, 2020	Prospective cohort	*EGFR*	82	*EGFR TKI*	PCR, tumor informed
Goldberg, 2018	Retrospective cohort	n/a	28	ICB	NGS, tumor informed
Han, 2022	Clinical trial	n/a	33	Chemoimmunotherapy	NGS, tumor agnostic
Joel, 2024	Prospective cohort	*EGFR*	66	*EGFR TKI*	PCR, tumor informed
Kwon, 2022	Prospective cohort	*ALK*	92	*EGFR TKIs*	NGS, tumor informed
Li, 2022	Retrospective cohort	*EGFR*	20	*EGFR TKIs*	NGS, tumor agnostic
Mack, 2022	Clinical trial	*EGFR*	106	*EGFR TKIs*	NGS, tumor informed
Mao, 2023	Retrospective cohort	*HER2*	50	HER2 TKIs	NGS, tumor agnostic
Murray, 2024	Prospective cohort	n/a	30	Chemoimmunotherapy or immunotherapy	NGS, tumor informed
Phallen, 2019	Retrospective cohort	n/a	28	*EGFR TKIs*	NGS, tumor agnostic
Provencio, 2021	Clinical trial	n/a	15	Chemoradiotherapy	PCR, tumor informed
Ricciuti, 2021	Prospective cohort	n/a	62	ICB	NGS, tumor informed
Romero, 2020	Prospective cohort	*EGFR*	22	*EGFR TKI*	NGS, tumor agnostic
Song, 2020	Clinical trial	n/a	248	Diverse	NGS, tumor agnostic
Soo, 2023	Clinical trial	*ALK*	291	*EGFR TKI*	NGS, tumor agnostic
Thompson, 2022	Prospective cohort	n/a	67	ICB	NGS, tumor agnostic
van der Leest, 2021	Prospective cohort	n/a	100	ICB	PCR, tumor informed
Vega, 2021	Clinical trial	n/a	200	ICB	NGS, tumor agnostic
Wang, 2018	Prospective cohort	*EGFR*	183	*EGFR TKI*	PCR, tumor informed
Wang, 2021	Clinical trial	*EGFR*	106	*EGFR TKI*	NGS, tumor agnostic
Weber, 2021	Prospective cohort	n/a	152	ICB	NGS, tumor agnostic
Yaung, 2022	Prospective cohort	n/a	92	Chemotherapy	NGS, tumor agnostic
Yu, 2022	Clinical trial	*METex14*	66	*MET TKI*	NGS, tumor agnostic
Zhang, 2024	Clinical trial	n/a	22	Chemoimmunotherapy	NGS, tumor agnostic
Pellini, 2023	Clinical trial	n/a	221	*EGFR* TKI and chemotherapy	PCR, tumor informed
Zheng, 2022	Retrospective cohort	*EGFR*	51	*EGFR TKI*	NGS, tumor agnostic
Zheng, 2024	Clinical trial	*ALK*	180	ALK TKI	NGS

Abbreviations: PCR, polymerase chain reaction; ICB, immune checkpoint blockade; TKI: tyrosine kinase inhibitor; n/a, not available; NGS, next generation sequencing

### Plasma ctDNA kinetics association with progression-free survival

**Figure 2.**
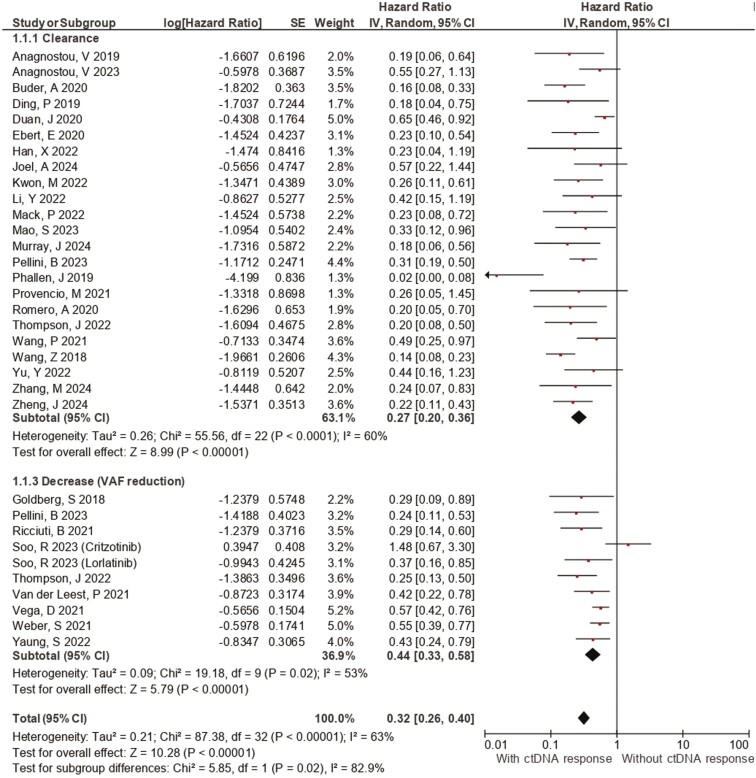
Forest plot of the association between plasma ctDNA kinetics and progression-free survival. Forest plots of the PFS hazard ratios regarding (a) ctDNA clearance, (b) *EGFR* clearance, and (c) ctDNA decrease. Squares are the effect size of the individual studies; diamonds, the summarized effect size; horizontal lines, upper, and lower borders of 95% CI; *P*-values < .05 are considered statistically significant.

Pooled results regarding PFS included 31 studies and revealed that patients with ctDNA decrease/clearance had improved outcomes (HR: 0.32 [0.26, 0.40], 95% CI, *I*² = 63%, *P* < .01). The subgroup of studies that reported ctDNA decrease (VAF reduction) also demonstrated its association with a better PFS (HR: 0.44 [0.33, 0.58], 95% CI, *I*² = 53%, *P* < .01). Notably, patients with ctDNA clearance (Figure 2) presented even greater benefits in PFS when compared with patients without clearance (HR: 0.27 [0.20, 0.36], 95% CI, *I*² = 53%, *P* < .01), with a statistically significant difference with the subgroup of studies that evaluated ctDNA decrease (*P* = .02). An increase in PFS was observed in patients with ctDNA decrease or clearance undergoing TT (HR: 0.34 [0.24, 0.46], 95% CI, *I*² = 61%, *P* < .01) and ICB (HR: 0.33 [0.24, 0.46], 95% CI, *I*² = 68%, *P* < .01). *EGFR*-mutant NSCLC patients who exhibited clearance of *EGFR* mutant clones in ctDNA demonstrated benefits in PFS (HR: 0.30 [0.22, 0.41], 95% CI, *I*² = 5%, *P* < .01). Tumor-agnostic assays showed a significant link between ctDNA clearance/decrease and PFS (HR 0.37, 95% CI [0.27, 0.50], *I*² = 65%, *P* < .01), with even stronger results for tumor-informed assays (HR 0.32, 95% CI [0.25, 0.42], *I*² = 43%, *P* < .01), but without statistically significant difference between the subgroups (Table 2).

**Table 2.** Summary of results comparing patients with ctDNA clearance/decrease and patients without molecular response.

Subgroups	Progression-free survival				Overall survival			
	HR (95% CI)	*P*	I2	Test for subgroup difference	HR (95% CI)	*P*	I2	Test for subgroup difference
*Treatment class*				*P* = .93				*P* = .27
ICB	0.33 [0.24, 0.46]	<.01	68%		0.32 [0.25, 0.41]	<.01	41%	
Targeted therapies	0.34 [0.24, 0.46]	<.01	61%		0.41 [0.28, 0.58]	<.01	0%	
*Assessment timepoint*				*P* = .76				*P* = .07
Timepoint < =4 weeks after baseline	0.29 [0.16, 0.53]	<.01	72%		0.32 [0.15, 0.36]	<.01	0%	
Timepoint > 4 weeks after baseline	0.33[0.25, 0.42]	<.01	63%		0.37 [0.29, 0.47	<.01	42%	
*Study design*				*P* = .11				*P* = .22
Clinical trial	0.40 [0.28, 0.57]	.01	68%		0.36 [0.29, 0.43]	<.01	43%	
Observational	0.28 [0.31, 0.36]	<.01	53%		0.32 [0.26, 0.40]	<.01	6%	
*Assay type*				*P* = .53				*P* = .17
Tumor agnostic	0.37 [0.27, 0.50]	<.01	65%		0.36 [0.29, 0.46]	<.01	25%	
Tumor informed	0.32 [0.25, 0.42]	<.01	43%		0.27 [0.19, 0.38]	<.01	55%	

Pooled results of PFS and OS according to different subgroups of studies. *P*-values < .05 are considered statistically significant.

Abbreviations: HR, hazard ratio; ICB, immune-checkpoint blockade.

### Plasma ctDNA kinetics association with overall survival

**Figure 3.**
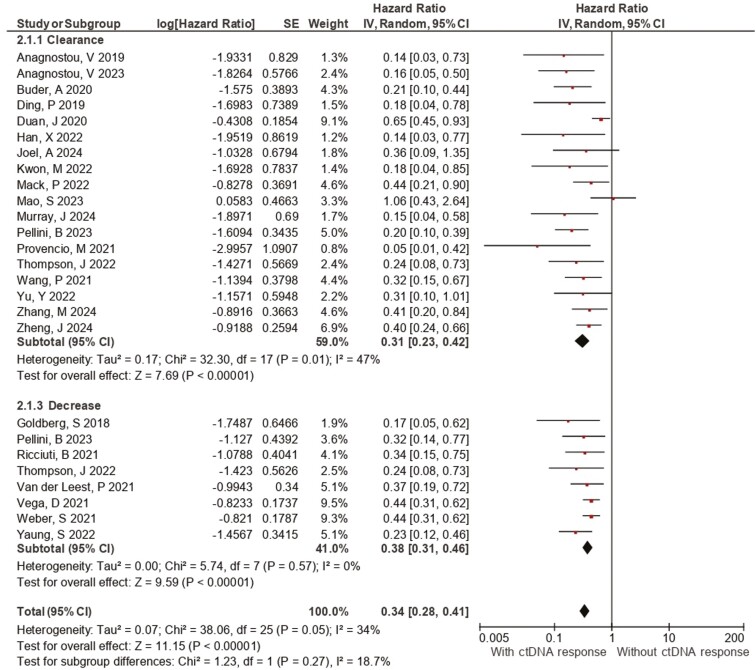
Forest plot of the association between plasma ctDNA kinetics and overall survival. Forest plots of the OS hazard ratios regarding (a) ctDNA clearance, (b) EGFR clearance, and (c) ctDNA decrease. Squares are the effect size of the individual studies; diamonds, the summarized effect size; horizontal lines, upper and lower border of 95% CI; *P* values < .05 are considered statistically significant.

Overall survival pooled results across 25 studies showed a significantly increased OS (Figure 3) in patients with ctDNA decrease/clearance (HR: 0.31 [0.23, 0.42], 95% CI, *I*² = 47%, *P* < .01). While ctDNA decrease was associated with a better OS (HR: 0.38 [0.31, 0.46], 95% CI, *I*² = 0%, *P* < .01), ctDNA clearance yielded even greater results (HR: 0.31[0.20, 0.47], 95% CI, *I*² = 59%, *P* < .01). The subgroup analysis according to treatment class demonstrated statically significant benefit of ctDNA clearance/decrease in patients undergoing TT (0.41 [0.28, 0.58], 95% CI, *I*² = 41%, *P* < .01) and ICB (0.32 [0.25, 0.41], 95% CI, *I*² = 24%, *P* < .0). The mutation-based clearance of *EGFR* mutant clones was associated with increased OS (HR: 0.30 [0.22, 0.41], 95% CI, *I*² = 5%, *P* < .01).

### Sensitivity analysis and meta-regression

Analysis regarding different study designs demonstrated that ctDNA clearance/decrease was associated with greater PFS both in observational studies (0.28 [0.21, 0.36], 95% CI, *I*² = 53%, *P* < .01) and clinical trials (0.40 [0.28, 0.57], 95% CI, *I*² = 68%, *P* < .01). Assessing ctDNA at baseline and up to 4 weeks (Table 2) after treatment showed a connection to improved PFS (HR 0.29, 95% CI [0.16, 0.53], *I*² = 72%, *P* < .01). Similar results were seen for assessments conducted after 4 weeks (HR 0.33, 95% CI [0.25, 0.42], *I*² = 63%, *P* < .01). In addition, meta-regression (Supplementary Figure S4) showed no significant association between the hazard ratios of PFS and NSCLC subtypes (*P* = .59), smoking status (*P* = .84), and sex (*P* = .61).

### Quality assessment

The visual inspection of the contour-enhanced funnel plot (Supplementary Figure S3) suggested that smaller and significant study results might be missing in the right portion of the plot, which could indicate the possibility of publication bias. However, the Egger test indicates the absence of asymmetry (*t* = −0.544, *P* = .59). All articles scored either moderate or low overall risk of bias on QUIPS checklist. A moderate risk of bias was attributed to some studies mainly due to confounding factors not properly analyzed. Results were summarized graphically with the traffic light plot and summary plot (Supplementary Figures S1 and S2).

## Discussion

This systematic review and meta-analysis findings demonstrated that advanced NSCLC patients undergoing systemic treatment presenting plasma ctDNA reduction or clearance derived significant benefits in OS and PFS compared to patients that did not present ctDNA clearance or decrease. These results were consistent across different therapeutic strategies, including TT and ICB. Patients presenting a ctDNA reduction or clearance in serial ctDNA sampling while undergoing systemic treatment showed improved survival outcomes; this association was seen regardless of the assay type and ctDNA assessment time points. Subgroup analysis showed the depth of molecular response was associated with better treatment outcomes and patients achieving ctDNA clearance demonstrated significant improved PFS when compared to ctDNA reduction. These findings highlight the importance of leveraging longitudinal ctDNA sampling as an early biomarker for timely detection of patients who are deriving a therapeutic response from systemic therapies and its role as a possible surrogate endpoint that may allow clinicians to take risk-adapted treatment decisions.

**Figure 4.**
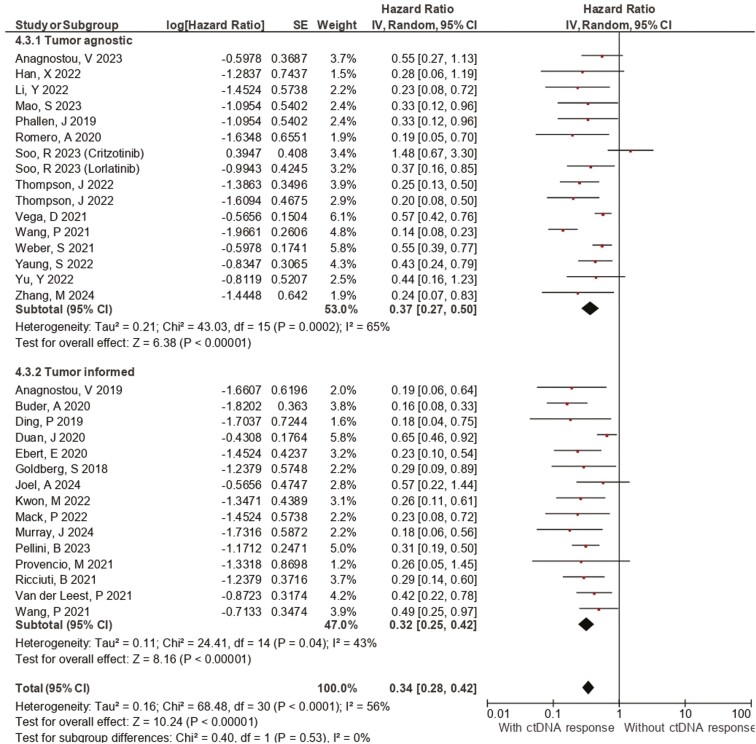
Forest plot of the association between plasma ctDNA kinetics and PFS according to assay type. Forest plots of the PFS hazard ratios regarding (A) ctDNA clearance, (B) EGFR clearance, and (C) ctDNA decrease. Squares are the effect size of the individual studies; diamonds, the summarized effect size; horizontal lines, upper and lower borders of 95% confidence interval; *P*-values < .05 are considered statistically significant.

Several methods are employed in the literature to summarize ctDNA levels and incorporate ctDNA characteristics for association with clinical outcomes.^58^ A key distinction is between tumor-informed and agnostic assays. Bespoke ctDNA assays use the specific somatic alterations of an individual’s tumor tissue, enhancing confidence in detecting cancer-derived mutations and subsequently potentially higher sensitivity accuracy, but this approach might be challenged by substantial mutational heterogeneity, few recurrently mutated genes, and difficulty in obtaining tumor tissue in patients to enable bespoke assays.^59,60^ Tumor-agnostic or tissue-free ctDNA approaches leverage a broad panel of common mutations without prior tumor knowledge, with the advantage of not needing tissue sampling but might have lower sensitivity accuracy.^61,62^ Pooled results from this meta-analysis (Figure 4) showed a significant correlation between ctDNA clearance/decrease and PFS using both tumor-agnostic (HR 0.37, 95% CI [0.27, 0.50]) and informed assays (HR 0.32, 95% CI [0.25, 0.42]). Interestingly, a similar association was reported in an exploratory analysis of the phase II randomized ctDNA-guided BR.36 trial, evaluating molecular response-adaptive immune-chemotherapy for patients with NSCLC, revealing 100% concordance in molecular response between the tumor-informed and agnostic approaches, while 90.9% of concordance was found regarding the cellular origin of variants.^32^

The substantial heterogeneity among assays and different timepoints employed in ctDNA analysis across studies is a barrier to establishing a standardized definition for ctDNA molecular response.^63^ Discrepancies in defining ctDNA reduction, often characterized as a decrease in mean variant allele fraction (VAF) or mutant allele frequency (MAF) of tumor-derived alterations over time, and clearance, referring to the complete elimination of circulating tumor load, across studies underscore the need to consider their varying implications for patient outcomes.^64^ For instance, a decrease of ctDNA >50% has been leveraged as a standard to characterize a molecular response in patients with advanced NSCLC^37,50,65^ and across other tumor histological types.^66-68^ Consistent with previous studies evaluating plasma ctDNA kinetics,^64^ our results indicate that ctDNA decrease was associated with increased PFS (HR: 0.44; 95% CI [0.33, 0.58]), and ctDNA clearance was associated with a more robust improvement in OS (HR: 0.27; 95% CI [0.20, 0.36]).

Mutation-driven ctDNA is another option for monitoring treatment response in advanced NSCLC, providing insights into the effectiveness of targeted treatments by tracking specific mutations such as *EGFR*, *ALK*, and *KRAS*.^69,70^ Monitoring the clearance of *EGFR*-clones in ctDNA has been described as a response predictor for EGFR tyrosine kinase inhibitors.^23,71,72^ Pooled results from our analysis revealed that patients with NSCLC who exhibited clearance of *EGFR* mutant clones in ctDNA demonstrated benefits in PFS (HR: 0.30 [0.22, 0.41], 95% CI) and OS (HR: 0.31 [0.19, 0.50], 95% CI). Furthermore, a previous study reported that patients diagnosed with advanced *KRAS*-mutant NSCLC undergoing therapy with adagrasib that presented clearance in *KRAS-*clones showed improved survival (14.1 months vs 8.7 months; *P* = .04; HR = 0.3).^44^ This evidence suggests that a mutation-based longitudinal ctDNA assessment may predict treatment response.

Optimal timing of on-treatment ctDNA analysis also lacks standardization and represents a hurdle to implementing longitudinal plasma ctDNA assessment in routine clinical practices.^73,74^ Evaluation-time bias and molecular kinetics variance, influenced by tumor shedding biology and different mechanisms across therapeutics, complicate the determination of a common optimal timepoint for plasma ctDNA sampling.^75,76^ Earlier studies suggest that for patients receiving ICB, optimal ctDNA analysis occurs earlier on treatment, between 4 and 9 weeks after treatment initiation, while patients undergoing TT or chemotherapy may benefit from earlier plasma monitoring.^77,78^ Earlier time points may better inform clinical decision-making but could compromise the sensitivity needed to accurately predict treatment response or disease progression.^79^ In line with previous findings,^58^ our meta-analysis results on the ctDNA decrease or clearance analyzed up to 4 weeks after treatment initiation yielded a discrete superior benefit (HR 0.23, 95% CI [0.15, 0.36]) to later timepoints analyses (HR 0.37, 95% CI [0.29, 0.47]) regarding OS.

While evolving evidence suggests early-on systemic therapy molecular response employing plasma ctDNA is prognostic, its integration into routine clinical practice faces significant challenges. One major barrier is the lack of prospective data supporting changes in treatment based on ctDNA results for patients diagnosed with advanced-stage NSCLC. Limited evidence demonstrates that altering therapy based on ctDNA kinetics improves patient outcomes in this setting. Ongoing clinical trials, such as the MERMAID-1 trial (NCT04385368), focus on ctDNA detection in early-stage NSCLC to assess minimal residual disease (MRD) after surgery and guide the use of adjuvant durvalumab plus chemotherapy.^80^ While this trial explores the role of ctDNA in guiding adjuvant therapy in resected patients, the evidence for real-time treatment change in advanced-stage cancer based on ctDNA kinetics remains insufficient. Prospective studies evaluating the use of ctDNA for therapeutic modulation in patients with advanced NSCLC are required for routine clinical implementation.

Our systematic review and meta-analysis provide informative results on the association between plasma ctDNA kinetics and survival outcomes in advanced NSCLC, however, some limitations warrant consideration. First, the included studies varied in their designs, patient populations, and methodologies, which may introduce heterogeneity and bias into our findings. Second, the reliance on published data limits our ability to access individual patient data, potentially limiting the depth of our analysis. Additionally, the possibility of publication bias, although assessed using funnel plots and statistical tests, cannot be completely ruled out. Despite these limitations, our study benefits from a comprehensive search strategy and the inclusion of a large number of studies, enhancing the robustness of our findings. Furthermore, the incorporation of sensitivity analyses with meta-regressions and subgroup analyses allowed for the exploration of potential sources of heterogeneity and provided valuable insights into the consistency of our results across different study designs. Overall, these findings contribute to a better understanding of the longitudinal plasma ctDNA kinetics as a prognostic biomarker for patients with advanced NSCLC undergoing systemic therapy, adding relevant data to the liquid biopsy field beyond plasma ctDNA use for cancer genotyping and treatment resistance.

## Conclusion

In our study, longitudinal or serial plasma ctDNA clearance or reduction was associated with improved survival outcomes in patients with advanced NSCLC undergoing TT and ICB. Our study results support the use of plasma ctDNA as an early endpoint in prospective clinical trials for further biomarker validation before incorporation in the clinical setting.

## Supplementary Material

oyae344_Suppl_Supplemental_Figures_S1-S4_Table_S1

## Data Availability

The data underlying this article will be share at a reasonable request by the corresponding author.

## References

[1] Thandra KC , Barsouk A, Saginala K, Aluru JS, Barsouk A. Epidemiology of lung cancer. Contemp Oncol. 2021;25:45-52. https://doi.org/10.5114/wo.2021.103829 PMC8063897 33911981

[2] Leiter A , Veluswamy RR, Wisnivesky JP. The global burden of lung cancer: current status and future trends. Nat Rev Clin Oncol. 2023;20:624-639. https://doi.org/10.1038/s41571-023-00798-3 37479810

[3] Chi SA , Yu H, Choi YL, et al Trends in survival rates of non-small cell lung cancer with use of molecular testing and targeted therapy in Korea, 2010-2020. JAMA Netw Open. 2023;6:e232002. https://doi.org/10.1001/jamanetworkopen.2023.2002 36929402 PMC10020884

[4] Ganti AK , Klein AB, Cotarla I, Seal B, Chou E. Update of incidence, prevalence, survival, and initial treatment in patients with non-small cell lung cancer in the US. JAMA Oncol. 2021;7:1824-1832. https://doi.org/10.1001/jamaoncol.2021.4932 34673888 PMC8532041

[5] Guo Q , Liu L, Chen Z, et al Current treatments for non-small cell lung cancer. Front Oncol. 2022;12:945102. https://doi.org/10.3389/fonc.2022.945102 36033435 PMC9403713

[6] Hendriks LE , Kerr KM, Menis J, et al; ESMO Guidelines Committee. Electronic address: clinicalguidelines@esmo.org. Non-oncogene-addicted metastatic non-small-cell lung cancer: ESMO Clinical Practice Guideline for diagnosis, treatment and follow-up. Ann Oncol. 2023;34:358-376. https://doi.org/10.1016/j.annonc.2022.12.013 36669645

[7] de Jager VD , Timens W, Bayle A, et al Future perspective for the application of predictive biomarker testing in advanced stage non-small cell lung cancer. Lancet Reg Health – Eur. 2024;38:100839. https://doi.org/10.1016/j.lanepe.2024.100839 38476751 PMC10928270

[8] Rotow J , Bivona TG. Understanding and targeting resistance mechanisms in NSCLC. Nat Rev Cancer. 2017;17:637-658. https://doi.org/10.1038/nrc.2017.84 29068003

[9] Yaung SJ , Woestmann C, Ju C, et al Early assessment of chemotherapy response in advanced non-small cell lung cancer with circulating tumor DNA. Cancers. 2022;14:2479. https://doi.org/10.3390/cancers14102479 35626082 PMC9139958

[10] Cheng ML , Lau CJ, Milan MSD, et al Plasma ctDNA response is an early marker of treatment effect in advanced NSCLC. JCO Precis Oncol. 2021;5:393. https://doi.org/10.1200/PO.20.00419 PMC8232122 34250387

[11] Cescon DW , Bratman SV, Chan SM, Siu LL. Circulating tumor DNA and liquid biopsy in oncology. Nat Cancer. 2020;1:276-290. https://doi.org/10.1038/s43018-020-0043-5 35122035

[12] Raez LE , Brice K, Dumais K, et al Liquid biopsy versus tissue biopsy to determine front line therapy in metastatic non-small cell lung cancer (NSCLC). Clin Lung Cancer. 2023;24:120-129. https://doi.org/10.1016/j.cllc.2022.11.007 36585341

[13] Park S , Olsen S, Ku BM, et al High concordance of actionable genomic alterations identified between circulating tumor DNA-based and tissue-based next-generation sequencing testing in advanced non-small cell lung cancer: the Korean Lung Liquid Versus Invasive Biopsy Program. Cancer. 2021;127:3019-3028. https://doi.org/10.1002/cncr.33571 33826761

[14] Febbo PG , Allo M, Alme EB, et al Recommendations for the equitable and widespread implementation of liquid biopsy for cancer care. JCO Precis Oncol. 2024;8:e2300382. https://doi.org/10.1200/PO.23.00382 38166232 PMC10803048

[15] Heitzer E , van den Broek D, Denis MG, et al Recommendations for a practical implementation of circulating tumor DNA mutation testing in metastatic non-small-cell lung cancer. ESMO Open. 2022;7:100399. https://doi.org/10.1016/j.esmoop.2022.100399 35202954 PMC8867049

[16] Duffy MJ. Circulating tumor DNA (ctDNA) as a biomarker for lung cancer: Early detection, monitoring and therapy prediction. Tumour Biol. 2024;46:S283-S295. https://doi.org/10.3233/TUB-220044 37270828

[17] Pascual J , Attard G, Bidard FC, et al ESMO recommendations on the use of circulating tumour DNA assays for patients with cancer: a report from the ESMO Precision Medicine Working Group. Ann Oncol. 2022;33:750-768. https://doi.org/10.1016/j.annonc.2022.05.520 35809752

[18] Tivey A , Church M, Rothwell D, Dive C, Cook N. Circulating tumour DNA—looking beyond the blood. Nat Rev Clin Oncol. 2022;19:600-612. https://doi.org/10.1038/s41571-022-00660-y 35915225 PMC9341152

[19] Casagrande GMS , Silva M de O, Reis RM, Leal LF. Liquid biopsy for lung cancer: up-to-date and perspectives for screening programs. Int J Mol Sci. 2023;24:2505. https://doi.org/10.3390/ijms24032505 36768828 PMC9917347

[20] Anagnostou V , Forde PM, White JR, et al Dynamics of tumor and immune responses during immune checkpoint blockade in non-small cell lung cancer. Cancer Res. 2019;79:1214-1225. https://doi.org/10.1158/0008-5472.CAN-18-1127 30541742 PMC6432636

[21] Mack PC , Miao J, Redman MW, et al Circulating tumor DNA kinetics predict progression-free and overall survival in EGFR TKI-treated patients with EGFR-mutant NSCLC (SWOG S1403). Clin Cancer Res. 2022;28:3752-3760. https://doi.org/10.1158/1078-0432.CCR-22-0741 35713632 PMC9444942

[22] Yamaguchi O , Kasahara N, Soda H, et al Predictive significance of circulating tumor DNA against patients with T790M-positive EGFR-mutant NSCLC receiving osimertinib. Sci Rep. 2023;13:20848. https://doi.org/10.1038/s41598-023-48210-5 38012343 PMC10682450

[23] Desai A , Vázquez TA, Arce KM, et al ctDNA for the evaluation and management of EGFR-mutant non-small cell lung cancer. Cancers. 2024;16:940. https://doi.org/10.3390/cancers16050940 38473302 PMC10930898

[24] Rolfo C , Mack P, Scagliotti GV, et al Liquid biopsy for advanced NSCLC: a consensus statement from the International Association for the Study of Lung Cancer. J Thorac Oncol. 2021;16:1647-1662. https://doi.org/10.1016/j.jtho.2021.06.017 34246791

[25] Page MJ , McKenzie JE, Bossuyt PM, et al The PRISMA 2020 statement: an updated guideline for reporting systematic reviews. BMJ. 2021;372:n71. https://doi.org/10.1136/bmj.n71 33782057 PMC8005924

[26] Hayden JA , van der Windt DA, Cartwright JL, Côté P, Bombardier C. Assessing bias in studies of prognostic factors. Ann Intern Med. 2013;158:280-286. https://doi.org/10.7326/0003-4819-158-4-201302190-00009 23420236

[27] DerSimonian R , Laird N. Meta-analysis in clinical trials. Control Clin Trials. 1986;7:177-188. https://doi.org/10.1016/0197-2456(86)90046-2 3802833

[28] Higgins JPT , Thompson SG. Quantifying heterogeneity in a meta-analysis. Stat Med. 2002;21:1539-1558. https://doi.org/10.1002/sim.1186 12111919

[29] Peters JL , Sutton AJ, Jones DR, Abrams KR, Rushton L. Contour-enhanced meta-analysis funnel plots help distinguish publication bias from other causes of asymmetry. J Clin Epidemiol. 2008;61:991-996. https://doi.org/10.1016/j.jclinepi.2007.11.010 18538991

[30] Egger M , Davey Smith G, Schneider M, Minder C. Bias in meta-analysis detected by a simple, graphical test. BMJ. 1997;315:629-634. https://doi.org/10.1136/bmj.315.7109.629 9310563 PMC2127453

[31] Zheng J , Wang T, Yang Y, et al Updated overall survival and circulating tumor DNA analysis of ensartinib for crizotinib-refractory ALK-positive NSCLC from a phase II study. Cancer Commun. 2024;44:455-468. https://doi.org/10.1002/cac2.12524 PMC11024683 38421881

[32] Anagnostou V , Ho C, Nicholas G, et al ctDNA response after pembrolizumab in non-small cell lung cancer: phase 2 adaptive trial results. Nat Med. 2023;29:2559-2569. https://doi.org/10.1038/s41591-023-02598-9 37814061 PMC10579094

[33] Buder A , Hochmair MJ, Setinek U, Pirker R, Filipits M. EGFR mutation tracking predicts survival in advanced EGFR-mutated non-small cell lung cancer patients treated with osimertinib. Transl Lung Cancer Res. 2020;9:239-245. https://doi.org/10.21037/tlcr.2020.03.02 32420063 PMC7225165

[34] Ding PN , Becker TM, Bray VJ, et al The predictive and prognostic significance of liquid biopsy in advanced epidermal growth factor receptor-mutated non-small cell lung cancer: a prospective study. Lung Cancer. 2019;134:187-193. https://doi.org/10.1016/j.lungcan.2019.06.021 31319980

[35] Duan J , Xu J, Wang Z, et al Refined stratification based on baseline concomitant mutations and longitudinal circulating tumor DNA monitoring in advanced EGFR-mutant lung adenocarcinoma under gefitinib treatment. J Thorac Oncol. 2020;15:1857-1870. https://doi.org/10.1016/j.jtho.2020.08.020 32916309

[36] Boysen Fynboe Ebert E , McCulloch T, Holmskov Hansen K, et al Clearing of circulating tumour DNA predicts clinical response to osimertinib in EGFR mutated lung cancer patients. Lung Cancer. 2020;143:67-72. https://doi.org/10.1016/j.lungcan.2020.03.020 32213382

[37] Goldberg SB , Narayan A, Kole AJ, et al Early assessment of lung cancer immunotherapy response via circulating tumor DNA. Clin Cancer Res. 2018;24:1872-1880. https://doi.org/10.1158/1078-0432.CCR-17-1341 29330207 PMC5899677

[38] Han X , Tang X, Zhu H, et al Short-term dynamics of circulating tumor DNA predicting efficacy of sintilimab plus docetaxel in second-line treatment of advanced NSCLC: biomarker analysis from a single-arm, phase 2 trial. J ImmunoTher Cancer. 2022;10:e004952. https://doi.org/10.1136/jitc-2022-004952 36600554 PMC9730395

[39] Joel A , Abarna R, Raju Chacko T, et al Analysis of the effect of baseline detection and early clearance of ct-DNA, on survival outcomes among patients with advanced EGFR-mutant non-small cell lung cancer. Klin Onkol. 2024;37:40. https://doi.org/10.48095/ccko202440 39183550

[40] Kwon M , Ku BM, Olsen S, et al Longitudinal monitoring by next-generation sequencing of plasma cell-free DNA in ALK rearranged NSCLC patients treated with ALK tyrosine kinase inhibitors. Cancer Med. 2022;11:2944-2956. https://doi.org/10.1002/cam4.4663 35437925 PMC9359877

[41] Mao S , Yang S, Liu X, et al Molecular correlation of response to pyrotinib in advanced NSCLC with HER2 mutation: biomarker analysis from two phase II trials. Exp Hematol Oncol. 2023;12:53. https://doi.org/10.1186/s40164-023-00417-y 37296463 PMC10251549

[42] Murray JC , Sivapalan L, Hummelink K, et al Elucidating the Heterogeneity of Immunotherapy Response and Immune-Related Toxicities by Longitudinal ctDNA and Immune Cell Compartment Tracking in Lung Cancer. *Clin Cancer Res*. 2024;30:389-403. https://doi.org/10.1158/1078-0432.CCR-23-1469 37939140 PMC10792359

[43] Phallen J , Leal A, Woodward BD, et al Early noninvasive detection of response to targeted therapy in non-small cell lung cancer. Cancer Res. 2019;79:1204-1213. https://doi.org/10.1158/0008-5472.CAN-18-1082 30573519 PMC6481620

[44] Paweletz CP , Heavey GA, Kuang Y, et al Early changes in circulating cell-free KRAS G12C predict response to adagrasib in KRAS mutant non-small cell lung cancer patients. Clin Cancer Res. 2023;29:3074-3080. https://doi.org/10.1158/1078-0432.CCR-23-0795 37279096 PMC10527102

[45] Provencio M , Majem M, Guirado M, et al Phase II clinical trial with metronomic oral vinorelbine and tri-weekly cisplatin as induction therapy, subsequently concomitant with radiotherapy (RT) in patients with locally advanced, unresectable, non-small cell lung cancer (NSCLC). Analysis of survival and value of ctDNA for patient selection. Lung Cancer. 2021;153:25-34. https://doi.org/10.1016/j.lungcan.2021.01.005 33453470

[46] Ricciuti B , Jones G, Severgnini M, et al Early plasma circulating tumor DNA (ctDNA) changes predict response to first-line pembrolizumab-based therapy in non-small cell lung cancer (NSCLC). J ImmunoTher Cancer. 2021;9:e001504. https://doi.org/10.1136/jitc-2020-001504 33771889 PMC7996662

[47] Romero A , Serna-Blasco R, Alfaro C, et al ctDNA analysis reveals different molecular patterns upon disease progression in patients treated with osimertinib. Transl Lung Cancer Res. 2020;9:532-540. https://doi.org/10.21037/tlcr.2020.04.01 32676317 PMC7354150

[48] Song Y , Hu C, Xie Z, et al; Written on behalf of AME Lung Cancer Collaborative Group. Circulating tumor DNA clearance predicts prognosis across treatment regimen in a large real-world longitudinally monitored advanced non-small cell lung cancer cohort. Transl Lung Cancer Res. 2020;9:269-279. https://doi.org/10.21037/tlcr.2020.03.17 32420066 PMC7225135

[49] Soo RA , Martini JF, van der Wekken AJ, et al Early circulating tumor DNA dynamics and efficacy of lorlatinib in patients with treatment-naive, advanced, ALK-positive NSCLC. J Thorac Oncol. 2023;18:1568-1580. https://doi.org/10.1016/j.jtho.2023.05.021 37295609

[50] Thompson JC , Carpenter EL, Silva BA, et al Serial monitoring of circulating tumor DNA by next-generation gene sequencing as a biomarker of response and survival in patients with advanced NSCLC receiving pembrolizumab-based therapy. JCO Precis Oncol. 2021;5:PO.20.00321. https://doi.org/10.1200/PO.20.00321 34095713 PMC8169078

[51] van der Leest P , Hiddinga B, Miedema A, et al Circulating tumor DNA as a biomarker for monitoring early treatment responses of patients with advanced lung adenocarcinoma receiving immune checkpoint inhibitors. Mol Oncol. 2021;15:2910-2922. https://doi.org/10.1002/1878-0261.13090 34449963 PMC8564646

[52] Vega DM , Nishimura KK, Zariffa N, et al Changes in circulating tumor DNA reflect clinical benefit across multiple studies of patients with non-small-cell lung cancer treated with immune checkpoint inhibitors. JCO Precis Oncol. 2022;6:e2100372. https://doi.org/10.1200/PO.21.00372 35952319 PMC9384957

[53] Wang Z , Cheng Y, An T, et al Detection of EGFR mutations in plasma circulating tumour DNA as a selection criterion for first-line gefitinib treatment in patients with advanced lung adenocarcinoma (BENEFIT): a phase 2, single-arm, multicentre clinical trial. Lancet Respir Med. 2018;6:681-690. https://doi.org/10.1016/S2213-2600(18)30264-9 30017884

[54] Wang P , Li Y, Lv D, et al Mefatinib as first-line treatment of patients with advanced EGFR-mutant non-small-cell lung cancer: a phase Ib/II efficacy and biomarker study. Signal Transduct Target Ther. 2021;6:1-9. https://doi.org/10.1038/s41392-021-00773-3 34719670 PMC8558340

[55] Weber S , van der Leest P, Donker HC, et al Dynamic changes of circulating tumor DNA predict clinical outcome in patients with advanced non-small-cell lung cancer treated with immune checkpoint inhibitors. JCO Precis Oncol. 2021;5:1540-1553. https://doi.org/10.1200/PO.21.00182 34994642

[56] Zheng J , Wang Y, Hu C, et al Predictive value of early kinetics of ctDNA combined with cfDNA and serum CEA for EGFR-TKI treatment in advanced non-small cell lung cancer. Thorac Cancer. 2022;13:3162-3173. https://doi.org/10.1111/1759-7714.14668 36193794 PMC9663669

[57] Pellini B , Madison RW, Childress MA, et al Circulating tumor DNA monitoring on chemo-immunotherapy for risk stratification in advanced non-small cell lung cancer. Clin Cancer Res. 2023;29:4596-4605. https://doi.org/10.1158/1078-0432.CCR-23-1578 37702716 PMC10643998

[58] Sanz-Garcia E , Zhao E, Bratman SV, Siu LL. Monitoring and adapting cancer treatment using circulating tumor DNA kinetics: current research, opportunities, and challenges. Sci Adv. 2022;8:eabi8618. https://doi.org/10.1126/sciadv.abi8618 35080978 PMC8791609

[59] Chan HT , Nagayama S, Otaki M, et al Tumor-informed or tumor-agnostic circulating tumor DNA as a biomarker for risk of recurrence in resected colorectal cancer patients. Front Oncol. 2023;12:1055968. https://doi.org/10.3389/fonc.2022.1055968 36776372 PMC9909342

[60] Stutheit-Zhao EY , Sanz-Garcia E, Liu ZA, et al Early changes in tumor-naive cell-free methylomes and fragmentomes predict outcomes in pembrolizumab-treated solid tumors. Cancer Discov. 2024;14:1048-1063. https://doi.org/10.1158/2159-8290.CD-23-1060 38393391 PMC11145176

[61] Carrasco R , Ingelmo-Torres M, Trullas R, et al Tumor-agnostic circulating tumor DNA testing for monitoring muscle-invasive bladder cancer. Int J Mol Sci. 2023;24:16578. https://doi.org/10.3390/ijms242316578 38068899 PMC10706140

[62] Honoré N , van Marcke C, Galot R, et al Tumor-agnostic plasma assay for circulating tumor DNA detects minimal residual disease and predicts outcome in locally advanced squamous cell carcinoma of the head and neck. Ann Oncol. 2023;34:1175-1186. https://doi.org/10.1016/j.annonc.2023.09.3102 37879442

[63] García-Pardo M , Makarem M, Li JJN, Kelly D, Leighl NB. Integrating circulating-free DNA (cfDNA) analysis into clinical practice: opportunities and challenges. Br J Cancer. 2022;127:592-602. https://doi.org/10.1038/s41416-022-01776-9 35347327 PMC9381753

[64] Al-Showbaki L , Wilson B, Tamimi F, et al Changes in circulating tumor DNA and outcomes in solid tumors treated with immune checkpoint inhibitors: a systematic review. J ImmunoTher Cancer. 2023;11:e005854. https://doi.org/10.1136/jitc-2022-005854 36792122 PMC9933752

[65] Nabet BY , Esfahani MS, Moding EJ, et al Noninvasive early identification of therapeutic benefit from immune checkpoint inhibition. Cell. 2020;183:363-376.e13. https://doi.org/10.1016/j.cell.2020.09.001 33007267 PMC7572899

[66] Kim ST , Cristescu R, Bass AJ, et al Comprehensive molecular characterization of clinical responses to PD-1 inhibition in metastatic gastric cancer. Nat Med. 2018;24:1449-1458. https://doi.org/10.1038/s41591-018-0101-z 30013197

[67] Zhang Q , Luo J, Wu S, et al Prognostic and predictive impact of circulating tumor DNA in patients with advanced cancers treated with immune checkpoint blockade. Cancer Discov. 2020;10:1842-1853. https://doi.org/10.1158/2159-8290.CD-20-0047 32816849 PMC8358981

[68] Forschner A , Battke F, Hadaschik D, et al Tumor mutation burden and circulating tumor DNA in combined CTLA-4 and PD-1 antibody therapy in metastatic melanoma - results of a prospective biomarker study. J ImmunoTher Cancer. 2019;7:180. https://doi.org/10.1186/s40425-019-0659-0 31300034 PMC6625062

[69] Iams WT , Mackay M, Ben-Shachar R, et al Concurrent tissue and circulating tumor DNA molecular profiling to detect guideline-based targeted mutations in a multicancer cohort. JAMA Netw Open. 2024;7:e2351700. https://doi.org/10.1001/jamanetworkopen.2023.51700 38252441 PMC10804266

[70] Kim S , Kim S, Kim SH, et al Clinical validity of oncogenic driver genes detected from circulating tumor DNA in the blood of lung cancer patients. Transl Lung Cancer Res. 2023;12:1185-1196. https://doi.org/10.21037/tlcr-22-912 37425402 PMC10326792

[71] Ahn M , Hartmaier R, Wu Y, et al FP16.03 early circulating-tumor DNA EGFR mutation clearance in plasma as a predictor of clinical outcomes in the AURA3 trial. J Thorac Oncol. 2021;16:S973-S974. https://doi.org/10.1016/j.jtho.2021.08.259

[72] Zhou C , Imamura F, Cheng Y, et al Early clearance of plasma EGFR mutations as a predictor of response to osimertinib and comparator EGFR-TKIs in the FLAURA trial. J Clin Oncol. 2019;37:9020-9020. https://doi.org/10.1200/jco.2019.37.15_suppl.9020

[73] Gouda MA , Janku F, Wahida A, et al Liquid biopsy response evaluation criteria in solid tumors (LB-RECIST). Ann Oncol. 2024;35:267-275. https://doi.org/10.1016/j.annonc.2023.12.007 38145866

[74] Saldanha EF , Nicolo E, Venetis K, et al The role of liquid biopsy as a catalyst for sustained progress in precision oncology—perspective of the young committee of the international society of liquid biopsy. J Liq Biopsy. 2024;5:100156. https://doi.org/10.1016/j.jlb.2024.100156 40027940 PMC11863974

[75] Bratman SV , Yang SYC, Iafolla MAJ, et al Personalized circulating tumor DNA analysis as a predictive biomarker in solid tumor patients treated with pembrolizumab. Nat Cancer. 2020;1:873-881. https://doi.org/10.1038/s43018-020-0096-5 35121950

[76] Samaille T , Bachelard CM, Coquan E, et al Impact of the timing of tumor assessments on median progression-free survival in clinical trials in advanced cancer patients. ESMO Open. 2022;7:1. https://doi.org/10.1016/j.esmoop.2021.100366 PMC8733185 34979424

[77] Moser T , Waldispuehl-Geigl J, Belic J, et al On-treatment measurements of circulating tumor DNA during FOLFOX therapy in patients with colorectal cancer. npj Precis Oncol. 2020;4:30. https://doi.org/10.1038/s41698-020-00134-3 33299124 PMC7666126

[78] Raja R , Kuziora M, Brohawn PZ, et al Early reduction in ctDNA predicts survival in patients with lung and bladder cancer treated with durvalumab. Clin Cancer Res. 2018;24:6212-6222. https://doi.org/10.1158/1078-0432.CCR-18-0386 30093454

[79] Osumi H , Shinozaki E, Yamaguchi K, Zembutsu H. Early change in circulating tumor DNA as a potential predictor of response to chemotherapy in patients with metastatic colorectal cancer. Sci Rep. 2019;9:17358. https://doi.org/10.1038/s41598-019-53711-3 31758080 PMC6874682

[80] Peters S , Spigel D, Ahn M, et al P03.03 MERMAID-1: a phase III study of adjuvant durvalumab plus chemotherapy in resected NSCLC patients with MRD+ post-surgery. J Thorac Oncol. 2021;16:S258-S259. https://doi.org/10.1016/j.jtho.2021.01.376

