# Supplementary material for: Plasma ctDNA kinetics as a predictor of systemic therapy response for advanced non-small cell lung cancer: a systematic review and meta-analysis
Source: Oncologist. 2025 Feb 25;30(2):oyae344. doi: 10.1093/oncolo/oyae344 (PMC11853598; doi:10.1093/oncolo/oyae344)
Supplement: oyae344_Suppl_Supplemental_Figures_S1-S4_Table_S1 [file oyae344_suppl_supplemental_figures_s1-s4_table_s1.docx]

**Supplemental Material**

**Supplemental Figure S1.** The quality assessment of the included studies - Risk of bias graph A

**
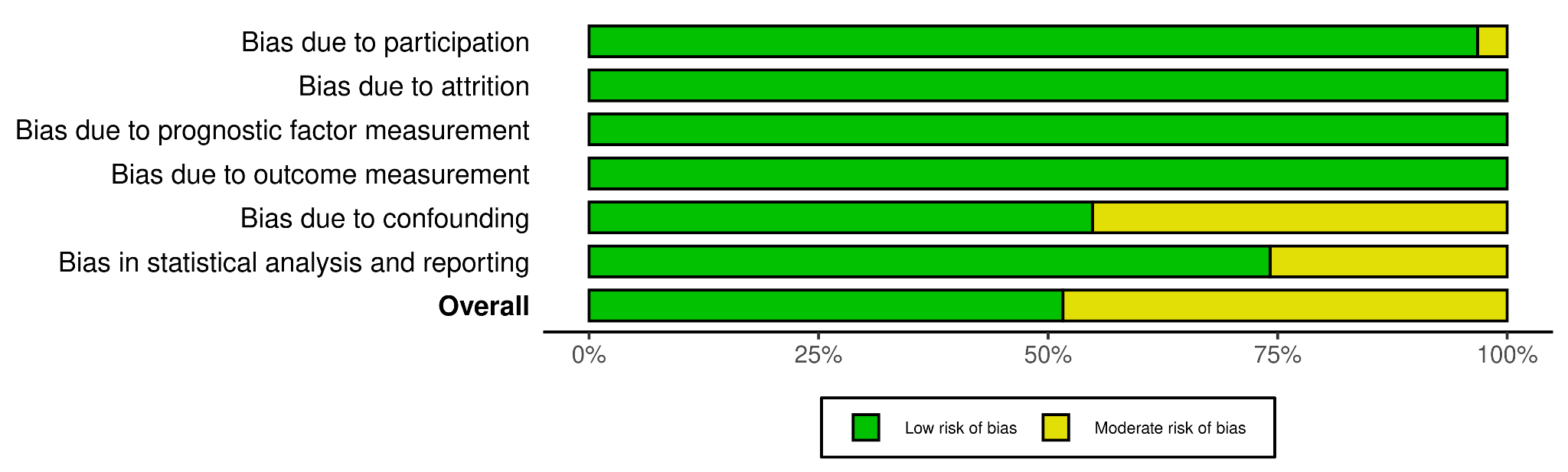
**

Chart of evaluated risk of bias in various studies according to the QUIPS checklist, showing the percentage of low (green), moderate (yellow) and high (red) risk across six domains.

**Supplemental Figure S2.** The quality assessment of the included studies - Risk of bias summary.

**
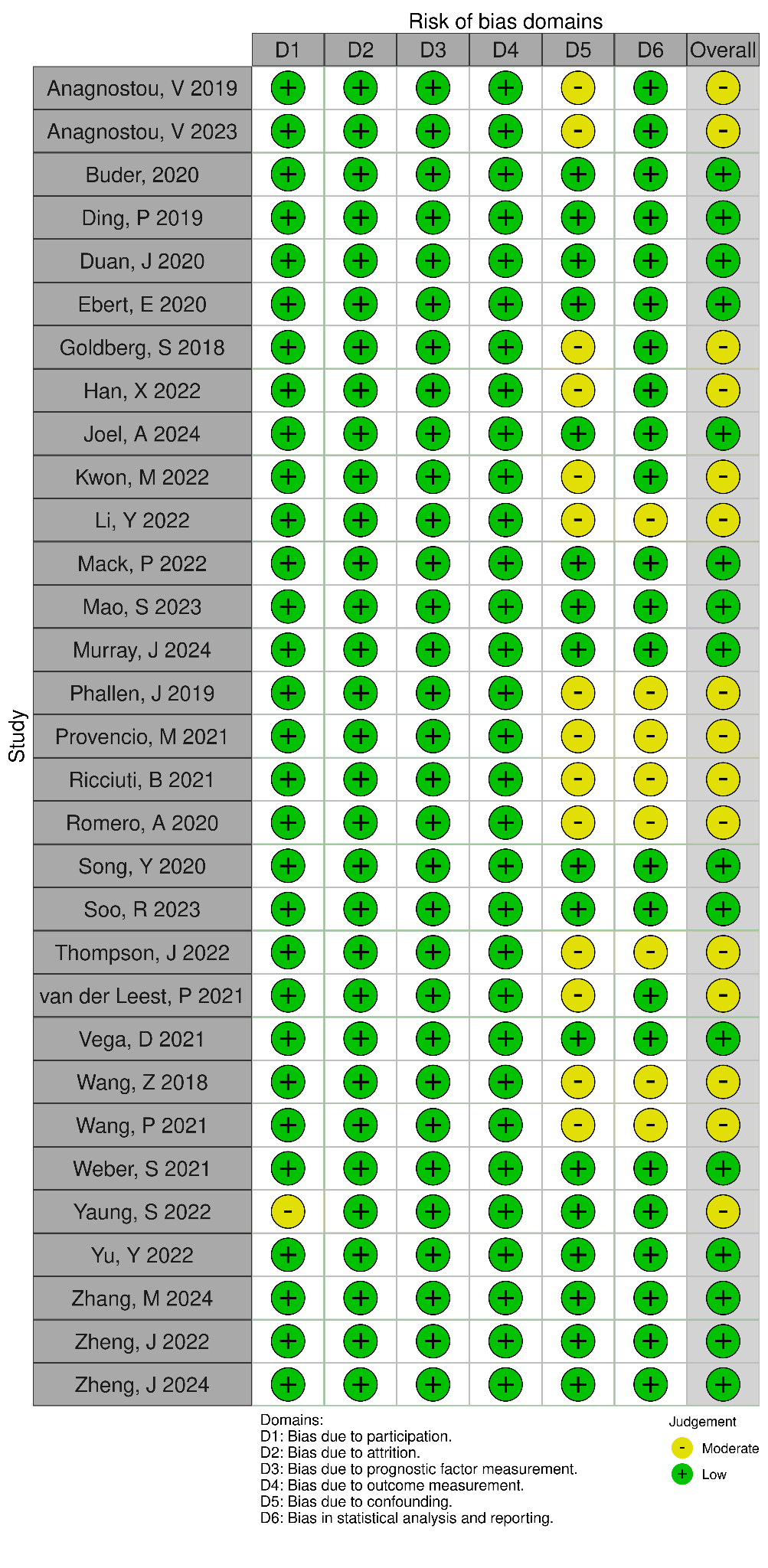
**

Traffic Light Plot of evaluated risk of bias in various studies according to the QUIPS checklist, with "+" indicating low risk, "-" indicating moderate risk, and "x" indicating high risk across six domains.

**Supplemental Figure S3-** Contour-enhanced funnel plot (overall survival)

**
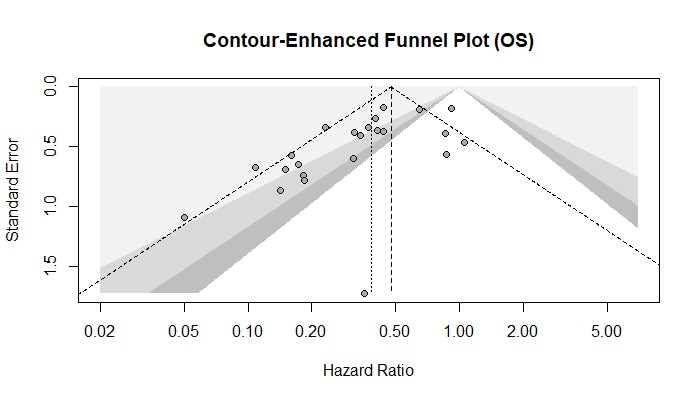
**

Figure S3 shows a contour-enhanced funnel plot for overall survival (OS), displaying hazard ratios against standard error. The plot suggests no significant publication bias.

**Supplemental Figure S4**- Bubble-plot of the meta regression between hazard ratio of PFS and the smoking status (A) or sex (B)**
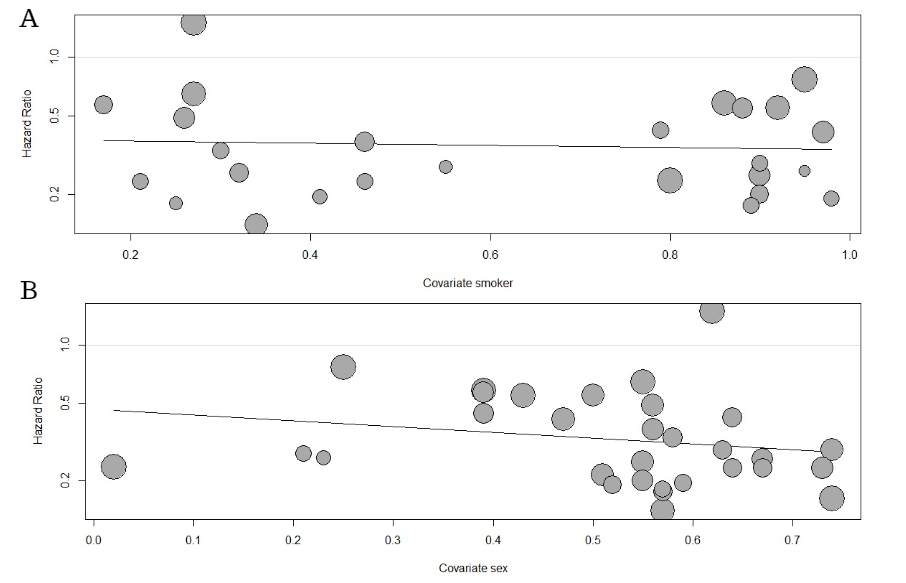
**

Figure S4 shows bubble plots for the meta-regression analysis between hazard ratios of progression-free survival (PFS) and smoking status (A) or sex (B). No significant associations were found between PFS hazard ratios and these covariates.

**Supplemental Table S1-** Additional baseline characteristics of included studies

| **Author, year** | **Total cohort** | **Patients with ctDNA clearance** | **Patients with ctDNA decrease** | **Cut-off values for molecular response (%VAF reduction)** | **Timepoint of ctDNA assessment** |
| --- | --- | --- | --- | --- | --- |
| Anagnostou, V 2019 | 24 | 9 | n/a | Undetectable | Prior to therapy and 4-8 weeks after initiation and additional time points until the time of disease progression |
| Weber, S 2021 | 152 | 8 | 100 | 50% | Baseline, after two cycles of ICI (4-6 weeks) |
| Thompson, J 2022 | 67 | 17 | 32 | 50% | Baseline, 9 weeks |
| van der Leest, P 2021 | 100 | 23 | 37 | 30% | Baseline, 4-6 weeks |
| Anagnostou, V 2023 | 50 | 14 | 15 |  | Baseline, 3 cycles |
| Vega, D 2021 | 200 | 63 | n/a | 50% | Baseline, 70 days |
| Ricciuti, B 2021 | 62 | 134 | 1728 | 98% | Baseline, after treatment initiation (median time to first assessment: 21 days) |
| Goldberg, S 2018 | 28 | 10 | 14 | 50% | Baseline, intervals of 2 or more weeks |
| Provencio, M 2021 | 15 | 89 | n/a | Undetectable | Baseline, 3 months |
| Murray, J 2024 | 30 | 6 | 11n/a | Undetectable | Baseline; at each treatment cycke |
| Zhang, M 2024 | 22 | 197 | n/a | Undetectable | Baseline, after cycle 1, after cycle 2 |
| Yaung, S 2022 | 92 | 18 | n/a | 50% | Baseline, 21-49 days after cycle 1, 21-49 days after cyle 2 |
| Han, X 2022 | 33 | 12 | n/a | ≤1 mutation detectable | Baseline, 6 week or after two cycles of treatment |
| Buder, A 2020 | 141 | n/a | n/a | <1 copy/mL | Baseline, 8 weeks |
| Ding, P 2019 | 28 | n/a | n/a | Undetectable | Baseline, 4 weeks |
| Duan, J 2020 | 180 | 100 | n/a | Undetectable | Baseline, every 8 weeks |
| Ebert, E 2020 | 82 | 33 | n/a | Undetectable | Baseline, 3-6 weeks |
| Joel, A 2024 | 66 | 17 | n/a | 99% | Baseline and 12-24 weeks |
| Kwon, M 2022 | 92 | 29 | n/a | Undetectable | Baseline, 8 weeks, disease progression |
| Li, Y 2022 | 20 | 715 | n/a | Undetectable | Baseline, 4 weeks, 12 weeks, disease progression |
| Mack, P 2022 | 106 | 45 | n/a | Undetectable | Baseline, 8 weeks, disease progression |
| Mao, S 2023 | 50 | 6 | n/a | 99% | Baseline, 40 days and 80 days |
| Phallen, J 2019 | 28 | 1214 | n/a | 98% | Baseline, 19 days, 47 days |
| Romero, A 2020 | 22 | n/a | n/a | Undetectable | Baseline, 3 months |
| Song, Y 2020 | 248 | 123 | n/a | Undetectable | Median: 95 days |
| Soo, R 2023 | 291 | 7953 | n/a | 50% | Baseline, week 4, week 24, and the end of treatment |
| Wang, Z 2018 | 183 | 1475 | n/a | Undetectable | Baseline, 8 weeks |
| Wang, P 2021 | 106 | 50 | n/a | Undetectable | Baseline, 6 weeks |
| Yu, Y 2022 | 66 | 1322 | n/a | Undetectable | Baseline, every 6 weeks in first year; every 12 weeks thereafter |
| Zheng, J 2022 | 51 | 30 | 31 | Undetectable | Baseline, after cycle 1 (2-4 months), after cycle 2 ( ≥4 months) |
| Zheng, J 2024 | 180 | 134 | 1728 | Undetectable | Baseline, 6 weeks |
| Pellini, B 2023 | 232 | 10 | 14 | Undetectable | Baseline, cycle 1, 2, 3, 4 and 8 weeks |

Table S1 summarizes additional baseline characteristics of the included studies, detailing total cohort size, the number of patients achieving ctDNA clearance or decrease, molecular response cut-off values (in terms of %VAF reduction), and the specific timepoints for ctDNA assessment across the studies.
